# Distribution of miRNA genes in the pig genome

**DOI:** 10.1186/s12863-015-0166-3

**Published:** 2015-01-30

**Authors:** Paulina Paczynska, Adrian Grzemski, Maciej Szydlowski

**Affiliations:** Department of Genetics and Animal Breeding, Poznan University of Life Sciences, Poland, Wolynska 33, 60-637 Poznan, Poland

**Keywords:** miRNA, Pig, Genomic location

## Abstract

**Background:**

Recent completion of swine genome may simplify the production of swine as a large biomedical model. Here we studied sequence and location of known swine miRNA genes, key regulators of protein-coding genes at the level of RNA, and compared them to human and mouse data to prioritize future molecular studies.

**Results:**

Distribution of miRNA genes in pig genome shows no particular relation to different genomic features including protein coding genes - proportions of miRNA genes in intergenic regions, introns and exons roughly agree with the size of these regions in the pig genome. Our analyses indicate that host genes harbouring intragenic miRNAs are longer from other protein-coding genes, however, no important GO enrichment was found. Swine mature miRNAs show high sequence similarity to their human and mouse orthologues. Location of miRNA genes relative to protein-coding genes is also similar among studied species, however, there are differences in the precise position in particular intergenic regions and within particular hosts. The most prominent difference between pig and human miRNAs is a large group of pig-specific sequences (53% of swine miRNAs). We found no evidence that this group of evolutionary new pig miRNAs is different from old miRNAs genes with respect to genomic location except that they are less likely to be clustered.

**Conclusions:**

There are differences in precise location of orthologues miRNA genes in particular intergenic regions and within particular hosts, and their meaning for coexpression with protein-coding genes deserves experimental studies. Functional studies of a large group of pig-specific sequences in future may reveal limits of the pig as a model organism to study human gene expression.

**Electronic supplementary material:**

The online version of this article (doi:10.1186/s12863-015-0166-3) contains supplementary material, which is available to authorized users.

## Background

MicroRNAs (miRNAs) are short (~22 nt) RNA sequences which play important role in posttranscriptional regulation of gene expression. Mature miRNA is part of active protein complex RISC (RNA - induced silencing complex) and inhibits translation of target transcript by binding to its 3′ UTR. MiRNAs are different from other classes of interfering RNA with its biogenesis, which was intensively investigated in human and mouse [1]. They are cut out from hairpin pre-miRNA (~70 nt) by enzyme Dicer in cytoplasm. Pre-miRNA is excised in nucleus from pri-miRNA - long transcript of miRNA gene - by enzyme Drosha. An individual pri-miRNA sequence may code multiple copies of pre-miRNA [2].

Human miRNA genes were found on all autosomes and X chromosome. A few predicted miRNA genes may be located on Y chromosome but they existence has not been confirmed [3]. Known miRNA genes occur within protein coding genes or within intergenic regions. Intragenic miRNAs are found within introns or exons. Location of miRNA genes in a genome can determine their expression and function. For example, it is hypothesised that an intragenic miRNA gene shares promoter sequence with its host gene [4]. MiRNA sequences exhibit high level of similarity among mammals, although some sequences in current nucleotide databases seem to be species specific. Although conservatism of mature miRNA sequences is well known, the conservatism in the location of miRNA genes was not systematically studied.

The pig (Sus scrofa) is one of the main sources of meet in human diet and is considered as potential donor of transplants. Due to its similarity to human in terms of anatomy, physiology, metabolism, genome and diet, the pig is important model organism [5]. Recent completion of swine genome may simplify the production of swine as a large biomedical model [6].

Regulatory function of a miRNA depends on the sequence of miRNA gene itself and on regulation of miRNA gene transcription. The regulation of a miRNA gene may be linked to localization of the gene in genome, and particularly to its position relative to protein-coding genes and CpG landscapes. Considerable differences in the location of orthologues miRNA genes between species would suggest that two orthologues miRNA, despite sharing high sequence similarity, may play their regulatory roles differently. To understand the limits of pig model and to direct future molecular studies in this paper we characterize location of swine miRNA genes and compare it to human and mouse data.

## Results and discussion

The number of known pig miRNA genes is relatively low when compared to human and mouse genomes (877 for pig vs. 4272 for human and 2009 for mouse, ver. Ensembl release 77), probably due to incomplete swine genome sequence and its annotation. The differences in the genome annotations may reflect lower interests and funding allocated so far for swine genome research. Recently, however, great progress has been made in comprehensive annotation of pig genome for noncoding RNAs [7]. We expect that some miRNA showing age-related activities (e.g. [8]) are not represented in pigs because older age groups are rarely sampled. Among the 877 swine miRNA sequences in Ensembl (rel. 77) only 273 sequences were included in miRBase (rel. 21) [9].

### Intra- and intergenic miRNA genes

The numbers of intergenic, intronic and exonic miRNA genes are presented in Table 1. In general, the proportions of different miRNAs are very similar in human and mouse, and different in the pig. Only 33% of porcine miRNA are intragenic vs 50% and 55% in human and mouse. Other in silico studies on human, mouse and chicken revealed that 41-47% of miRNAs overlap with protein-coding genes [10]. This disparity between the pig and other studied species may result from the lower number of available porcine miRNA sequences rather than being a particular feature of pig genome. However, the question arises why the statistics are still so different for the pig despite the fact that considerable number of porcine miRNA genes are already available (N = 877). First, it can be easily observed that the inclusion of different miRNA types in the database in period of time is not in proportion to their actual occurrences, probably being a result of particular alterations made in a pipeline used to build newer releases. For example, Ensembl release 77 (October 2014) includes 306 more mouse intragenic sequences and only 53 more intergenic miRNA when compared to release 70 (January 2013), whereas the proportion of these types is estimated to be 1:1, approximately. Second, the pig miRNAs were identified only in a few experiments limited to several tissues and age groups. In such case, some clusters of miRNAs having similar location and expression patterns can be strongly overrepresented. In consequence, when a database is in early stage, like in case of swine miRNAs, such comparisons between species must be treated with great caution. Third, it is also possible that some intragenic miRNA were misclassified as intergenic because of incomplete and imprecise annotation of protein-coding genes based on the direct evidence from known transcripts. The number of known transcripts per protein coding gene is only 1.2 in pig (average transcript length 31.3 kbp) compared to 6.9 in human (average 38.6 kbp).

**Table 1:** **Number of miRNA and host genes (Ensembl, release 77)**

	**Pig**			**Human**			**Mouse**		
	**N**	**%**	**%**	**N**	**%**	**%**	**N**	**%**	**%**
**miRNA total**	877	100.0		4272	100.0		2009	100.0	
Intergenic	587	66.9		2132	49.9		900	44.8	
Intragenic	290	33.1	100.0	2140	50.1	100.0	1109	55.2	100.0
Host strand	186		64.1	1482		69.3	825		74.4
Intron	166		57.2	1282		59.5	710		64.0
Exon	20		6.9	200		9.3	115		10.4
Opposite strand	95		32.8	550		25.7	244		22.0
Intron	71		24.5	485		22.7	197		17.8
Exon	24		8.3	65		3	47		4.2
With multiple hosts	9		3.1	108		5	40		3.6
**Hosts total**	272	100.0		1887	100.0		930	100.0	
miRNA strand	173	63.6	100.0	1201	63.6	100.0	646	69.5	100.0
With intronic miRNA	155		89.6	1011		84.2	540		83.6
With exonic miRNA	18			190			106		
Opposite strand	82	30.1	100.0	449	23.8	100.0	216	23.2	100.0
With intronic miRNA	59		72.0	385		85.7	175		81.0
With exonic miRNA	23			64			41		
With multiple miRNAs	17	6.3		237	12.6		68	7.3	

We examined whether the number of intragenic miRNA genes are proportional to the total relative size of protein-coding genes in genome (% of total represented bp: pig 25%, human 39%, mouse 25%). The proportion of all intragenic miRNAs (including both host oriented and opposite strand miRNAs: pig 33%, human 50%, mouse 55% of all miRNAs) was higher than percentage of genomic DNA occupied by all protein coding genes. These numbers suggest that new miRNAs evolve faster in introns or exons than within intergenic regions. However, when we excluded all intragenic miRNAs located on opposite strand, this tendency was not so obvious. In this case, the percentage of remaining host-oriented miRNAs among all miRNA genes (pig 21%, human 35%, mouse 41%) was roughly what could be expected given the relative size of protein-coding genes in pig (21 vs 25%) and human (35 vs 39%), but it was still high in mouse (41 vs 29%).

It is suggested that an intragenic host-oriented miRNA may share host’s promoter, whereas miRNA on alternative strand is unlikely to utilize host’s regulatory mechanism [11]. Studies of mammals’ genomes showed significant overrepresentation of miRNA genes in introns of protein coding genes and higher proportion of intronic miRNA genes on the sense strand [12]. However, taking together these statistics we noticed that intragenic miRNAs that potentially utilize their hosts’ promoters do not emerge in genome more often than miRNAs in intergenic regions. Therefore, the thesis that intragenic region is a ‘sweet spot’ for the emergence of novel miRNAs because the prior evolution of a new promoter unit is not required is not supported by our analysis of three mammal genomes. Moreover, if indeed the lack of protein-coding promoters constitutes a limit for new miRNAs to arise, the number of intergenic miRNAs would be low. However, the number of intergenic miRNAs is close to that expected by chance. Roughly 10% of intragenic miRNAs are located in exons. Again, this is what can be expected by chance given that annotated exons in database represent about 5% of protein-coding genes in pig and 11% in human and mouse. The fact that exonic regions do not decrease the number of miRNAs is intriguing because a miRNA sequence needs to be self-complementary to form functional stem-and-loop structure.

**Figure 1:**
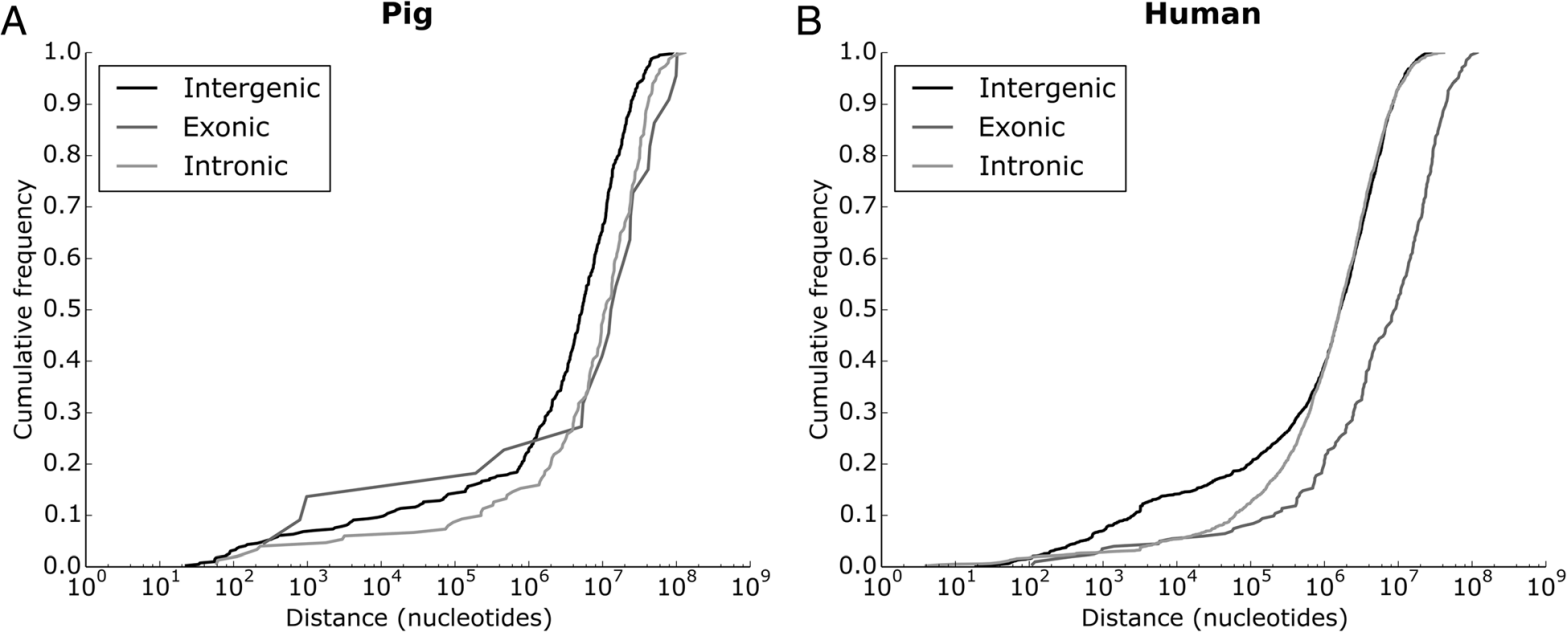
**Cumulative distance distribution of miRNA genes in pig (A) and human (B).** For each type of the described miRNA genes (intergenic, intronic, exonic) the distances (in nucleotides) between every two same-chromosome same-strand successive miRNA genes were obtained from Ensembl (ver. 77). Distance is drawn on a logarithmic scale.

It was demonstrated that certain structured non-coding RNAs in the pig genome form clusters based on genomic positions. With cutoff of 10,000 nt different ncRNA genes form numerous clusters, mostly pairs [7]. Here, we observed that different types of porcine miRNA genes show very similar tendency to occur in clusters (Figure 1). This result is in contrast to human genome, where intergenic miRNA genes show markedly higher tendency to be clustered than intronic and exonic miRNA genes. However, the cumulative distance distributions of intergenic miRNA genes are very similar in these three species. Clustering of miRNA genes in human genome was characterized in detail by [13]. It was found that ‘short-range’ clustering is strongly linked to ‘same-strand’ clustering, which in turn is more likely to be linked to policistronic transcription. Our analysis show that policistronic transcription may more likely occur in intergenic miRNA genes than for intragenic miRNA genes. On other hand, the increased probability of policistronic transcription in intergenic regions may be species-specific.

### Host genes

The 290 known pig intragenic miRNA genes are localized in 272 protein-coding host genes (Table 1). The hosts harbouring more than one miRNA sequence are rare. We analysed all 182 porcine host genes that include at least one host-oriented miRNA gene in intronic or exonic region. We observed that a random host gene is usually much longer than a random protein coding gene in a genome (2.6 - 4.3 fold longer) and contains more exons (1.6 - 2 times more). Typically, exons occupy only small portion of host gene: 1.9 - 5.6% of its length compared to 4.7 - 11% in random gene, therefore the difference between hosts and random genes are mainly due to intronic regions. The increased number of exons, however, also translates to transcript size. The average length of transcripts from a swine host gene was higher than for a random gene: 75′839 nt (N = 401) in a host gene and 31′216 nt in a random gene (N = 26′712), respectively. Similar results were obtained for human: the average length of transcripts from human host genes was 80′241 nt (N = 18′588) compared to 38′617 nt for random gene (N = 153′638).

The DAVID algorithm revealed that host genes in human genome (N = 1887) more often code for coiled-coil protein structures than random genes (p-value = 1.7 × 10^−8^, fold enrichment 1.5, 200 hosts involved). Among various biological functions coiled-coil structures are involved in gene regulation and form fibrous proteins, which are expected to be longer. We observed that 259 hosts are more often expressed in epithelium (p-value = 1.1 × 10^−10^, fold enrichment 1.5). Therefore the enrichment for coiled-coil structure may be connected with increased length of average host gene. Although the enrichment is significant the two features constitute a minority within all host genes. We also identified 90 transcription factors significantly enriched in regulation of the set of human hosts. Next we narrowed down our analysis of human hosts to those that harbour miRNA in their exons on same strand (N = 190 hosts). The DAVID indicated that this set is enriched for RNA binding (p-value = 2.6 × 10^−6^, fold enrichment 3.8, 18 hosts involved) and regulation of transcription (p-value = 8.3 × 10^−4^, fold enrichment 1.8, 30 hosts involved). Other important gene-term enrichment included the coiled-coil and epithelium again. There was no common feature shared by the majority of this set of human hosts, except that 72 hosts were described as phosphoproteins (p-value = 2.2 × 10^−5^, fold enrichment 1.5). Eighteen transcription factors were enriched.

The above enrichment analysis was performed for the identified host genes in human genome with the tool designed for human genes. We found that location of intragenic miRNA is mostly conservative between pig and human, and therefore, pig hosts often have their orthologues counterparts in the human genome. Consequently, the enrichment analysis above should approximate the situation in the pig. Nevertheless, with the increasing importance of the pig as model organism, there is a need for appropriate tools better suited for swine genome.

It must be noted, however, that we did not distinguish between hosts that share their promoters with internal miRNA and other hosts harbouring miRNAs that have their own promoters. Such classification is not yet possible. In future it may be possible to distinguish between these two types by the use of gene expression profiling. For the current analysis we attempted to classify our hosts based on phylogeny data on miRNAs. It was shown that phylogenetically old intragenic miRNAs are more coexpressed with their hosts than young ones [14]. This observation suggests that phylogenetically old miRNAs use their hosts’ promoters more often than phylogenetically young miRNAs. We identified 41 human hosts harbouring conservative (old) miRNAs (information on evolutionary conservation status was downloaded from TargetScan, we considered only conservation status of type II – highly conserved miRNAs). Again, this group show enrichment for the coiled-coil structure (p-value = 2.5 × 10^−3^, fold enrichment 2.8, 13 hosts involved) and also alternative splicing (p-value = 9.3 × 10^−3^, fold enrichment 1.6, 24 hosts involved), which both may be connected with gene length.

Assuming that an intragenic miRNA gene shares regulatory mechanism with its protein-coding host gene, we further studied the distribution of CpG islands in the 5′flanking regions of porcine hosts. High frequency of CpG islands would indirectly suggest involvement of intragenic miRNA genes in the control of developmental processes. However, we found no difference in the distribution of CpG islands within 5′ flanking region between swine host and random gene. Twenty three percent of host genes and 22% of all protein coding genes had at least one predicted CpG island in 5′ flanking region and the average number of CpG islands was 1.3. We calculated very similar statistics for human hosts (23% with CpG, 1.3 CpG island per gene). We also observed that porcine host genes have similar codon usage statistic to random gene (average Nc statistics: 53).

In vertebrates CpG islands are properties of different types of promoters [15]. It was observed that genes showing tissue-specific expression in adult peripheral tissues have mostly no CpG islands, whereas genes showing broad expression through organismal cycle have CpG islands. Large CpG islands are feature of promoters of differentially regulated genes, regulators in multicellular development and differentiation. Our results on the distribution of CpG islands in the close vicinity of genes hosting miRNAs are in agreement with the general observation that intragenic miRNAs as other noncoding RNAs play roles in a wide variety of biological mechanisms.

Our analysis suggests that a host protein-coding gene harbouring a miRNA gene is not very different from other protein-coding genes in the three studied mammalian genomes. Together with our observation on even distribution of miRNAs in intronic, exonic and intergenic regions, the analyses of host genes support a view that a protein-coding gene becomes a host gene by random acquisition of miRNA locus. The rate of acquisition is independent of protein-coding gene, except that longer protein-coding genes have a higher chance of hosting a miRNA gene. The distribution of miRNAs in mammalian genome is roughly random (except clusters of miRNAs) with no genomic landscapes and clear connection to particular sets of protein-coding genes. We can further speculate that if intragenic miRNAs are coexpressed with host genes and this coexpression model is correct, such mechanism of posttranscriptional regulation would not be limited to particular metabolic pathways. If there are functional links between intragenic miRNAs and their hosts, the current comparison of hosts and random genes suggests that intragenic miRNAs are players in regulatory mechanisms for genes showing different pattern of expression.

As most porcine protein-coding genes, including 272 host genes, have their orthologues in the human genome, the analyses of human hosts genes described here can be considered as an indirect examination of porcine host genes through their better annotated human orthologues.

### Phylogeny evidence for miRNA genes

In general, the level of phylogeny evidence is markedly lower for miRNA than for protein coding genes (Table 2). Probability for a swine miRNA gene to have a human ortholog (of any type) in the database is 45% compared to 86% for a random protein-coding gene. It is possible that for many miRNA genes the existing orthologues sequences have not been detected yet. However, when we compared better annotated genome of mouse (2009 miRNA genes) to human data we observed that the proportion of orthologues pairs within miRNA genes is even lower (16%). Hence, we can expect that after improving annotation of the genome of the pig in near future, still a significant part of the pig miRNA genes will have no orthologs in human genome.

**Table 2:** **The level of phylogenic evidence for porcine miRNA and protein-coding genes**

**Comparison**	**One-to-one ortholog**	**Other type ortholog**	**No ortholog**
**Pig to human**			
miRNA genes (N = 877, 100%)	32%	13%	55%
Protein-coding genes (N = 21607, 100%)	59%	27%	14%
**Pig to mouse**			
miRNA genes (N = 877, 100%)	27%	10%	63%
Protein-coding genes (N = 21607, 100%)	58%	28%	13%
**Mouse to human**			
miRNA genes (N = 2009, 100%)	15%	1%	84%
Protein-coding genes (N = 22187, 100%)	71%	12%	17%

Percentage of the porcine genes in the Ensembl Compara database having one-to-one or other type orthologs in human and mouse genomes. The mouse-to-human phylogeny was included for comparison (Ensembl release 77).

Current view is that miRNA genes are continuously being added to metazoan genomes through geological time [16]. It was observed that acquisition and fixation of miRNAs in various animal groups correlates both with the hierarchy of metazoan relationships and with the non-random origination of metazoan morphological innovations through geologic time [17]. Because phylogenetic distance between human and mouse is considered lower than between human and pig, the proportion of shared miRNAs between human and pig should not exceed that between human and mouse. Interestingly, there are considerable differences in the number of species-specific miRNA genes in the three genomes. About 53% of the pig miRNA genes have no ortholog in other species included in the Compara database (rel. 77), whereas for human and mouse the percentage of unique miRNA genes is higher (90% and 84%, respectively).

Considering swine miRNA genes having orthologs in human genome, we observed that 71% of the shared miRNAs genes were one-to-one orthologs. However, similar level was calculated for protein-coding orthologs (69%). Comparison between mouse and human also showed that orthology between miRNA genes can be defined as good as for protein-coding genes (94% and 86% pairs, respectively, are one-to-one type) despite large difference in sequence length between miRNA and protein-coding genes. It must be noted, however, that we did not consider uncertainty in the topology of individual phylogenetic trees in the Compara database.

### Conservatism of miRNA orthologs

We aligned 284 pig sequences coding for pre-miRNAs (70-100 nt) with their human one-to-one orthologs. Mean percentage of identity from local alignment was 93% (range 61% - 100%). Similar values were calculated for pig-to-mouse (N = 235 pairs, identity 92%) and mouse-to-human (N = 297 pairs, identity 92%) alignments. When we aligned only intragenic miRNA genes located in hosts being one-to-one orthologues the mean identity was not higher. However, sequence identity decreases when orthology status is less certain. For example, the percentage identity for ‘apparent’ one-to-one orthologues is only 80% (65-100%) between pig and human, 82% (55-100%) between pig and mouse and 76 (59-94%) between mouse and human.

Next we aligned sequences coding for mature miRNA (~22 nt, we included all sequences having accession number in miRBase). Our comparison confirmed high conservatism among mature miRNA sequences [18]. The mean identity between pig and human was 97.8% (range 78.3% - 100%, 178 pairs), between pig and mouse was 96.8% (range 66.7% - 100%, 171 pairs), and between mouse and human 97.8% (69.2% - 100%, 283 pairs).

### Location of miRNA genes

**Figure 2:**
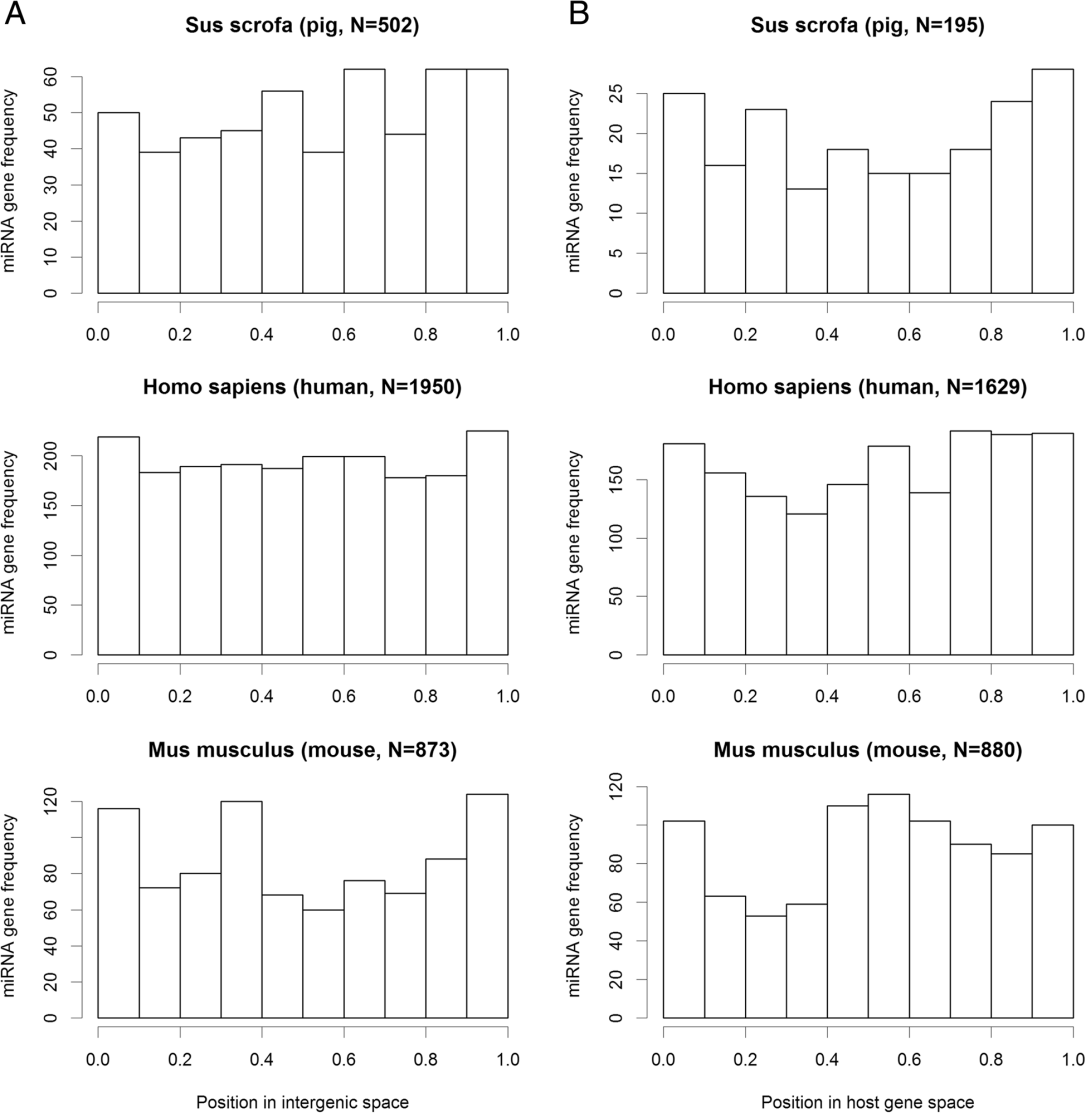
**Distribution of location of miRNA genes in the genomes of pig, human and mouse. A)** Positions of miRNA genes in intergenic space. The space between flanking protein-coding genes was standardized to 1 and the position of each intergenic miRNA gene was mapped on the standardized space. Value close to zero at x-axis indicates that miRNA is situated closer to 5′ flanking gene and values closer to 1 indicate localization of miRNA close to 3′ flanking gene. **B)** Positions of intragenic miRNA genes in gene space. The space between start and end of a host gene was standardized to 1 and the position of each intragenic miRNA gene was mapped on the standardized space. Value close to zero at x-axis indicates that miRNA is situated closer to start of host gene and value closer to 1 indicates localization of miRNA close to end of this host gene. The number of miRNA genes analyzed is given in parentheses.

We investigated whether there is any tendency in localization of a miRNA in intergenic space. For each intergenic miRNA in pig, human and mouse genomes we searched 10^7^ bp regions in both directions (5′ and 3′) for existence of protein coding genes. The threshold of 10^7^ was determined because in the human genome 100% of the pairwise distances between same-strand protein-coding genes are below 10^7^ nucleotides [13]. We chose the closest gene in 5′ flanking region of miRNA sequence and separately the nearest gene in 3′ flanking region (note, miRNA having no flanking gene within this distance were excluded). Average distance to 5′ flanking gene in pig genome was 0.464 Mb (mega base pairs) and average distance to 3′ flanking gene was 0.504 Mb (in human genome: 0.633 Mb and 0.663 Mb respectively; in mouse genome: 0.487 Mb and 0.514 Mb). Thus, these results show no tendency in positions of intergenic miRNA genes. To describe the positions of intergenic miRNAs in greater detail we present bar plots showing number of miRNAs in particular position in standardized intergenic space (Figure 2A). Again, the plots show no clear tendency in miRNA localization within intergenic space. For human with the highest number of known miRNA genes the distribution is almost uniform. This lack of tendency in miRNA position suggests that intergenic miRNA are regulated independently from their flanking protein coding genes. Whether this is true particularly for the miRNAs that are most adjacent to protein coding genes must be further verified.

To visualize the location of intragenic miRNA genes within their host protein-coding genes we present additional bar plots for the same three species (Figure 2B). The plots show no consistent (common for all species) tendency in intragenic miRNA localization. If we consider only human (with highest number of know miRNA genes), we can observe small tendency towards location of miRNA genes in both terminal fragments of host gene. Similar tendency could be observed in pig.

Hinske et al. [19] found that 65.5% of human host genes had miRNAs in the first five introns, however, this observation does not necessarily mean a bias in miRNA position toward 5′ end of host gene. Almost 93% of human host genes harbouring miRNAs sequences have at least 5 introns, whereas only 84% includes 6 or more introns. Thus, a priori, the chance of finding miRNA genes in a few first introns is higher than for subsequent introns. Interestingly, mouse and rat (Additional file 1: Figure S1) seem to have alternative tendency of miRNAs location, with most intragenic miRNAs genes localized in a central part of host gene.

### Conservatism of the location of miRNA genes

We defined a position of each miRNA gene in relation to protein-coding genes and compared the positions between species. First, we checked whether pig intragenic miRNA genes are harboured by same hosts as their miRNA orthologs in human genome. As expected, for most pig intragenic miRNA genes (72%) the comparison to human genome was impossible due to missing or incomplete information on phylogenetic relation between genes. Note that in order two compare positions between two species both porcine miRNA and host gene need to have one-to-one orthologs in human genome. Within the remaining 82 informative comparisons we encountered 16 miRNA genes with different location between species. Six orthologs were intergenic in human and other 10 were located in non-orthologues hosts (Additional file 2: Data S1). These dissimilar locations could be partly explained by the incorrect annotation of protein-coding hosts, which may be longer than described based on available transcripts. The comparisons between pig and mouse (64 pairs) and between mouse and human (140 pairs) also revealed rare individual differences between species. In most such cases, an orthologues miRNA genes were found in non-orthologues hosts (pig v. mouse: 2 cases; mouse v. human: 13 cases).

Next we compared positions of intergenic miRNA genes between species. Again, most miRNA genes could not be compared due to unknown or ambiguous phylogeny. Note that in order to detect dissimilar location of miRNA between species the two protein coding genes flanking the miRNA locus must both have orthologs in other species. Within 55 informative cases in the pig-to-human comparison we observed 12 dissimilar locations (9 human orthologues miRNA genes were found in different intergenic space, whereas 3 others were intragenic) (Additional file 2: Data S1). Further, within 54 informative cases in the pig-to-mouse comparison we observed 6 dissimilar locations (4 in different intergenic region and 2 intragenic). The mouse-to-human comparison (98 informative cases) revealed only 5 such dislocations, therefore, it is possible that some of the pig-human dissimilarities stem from incorrect genome annotation.

**Figure 3:**
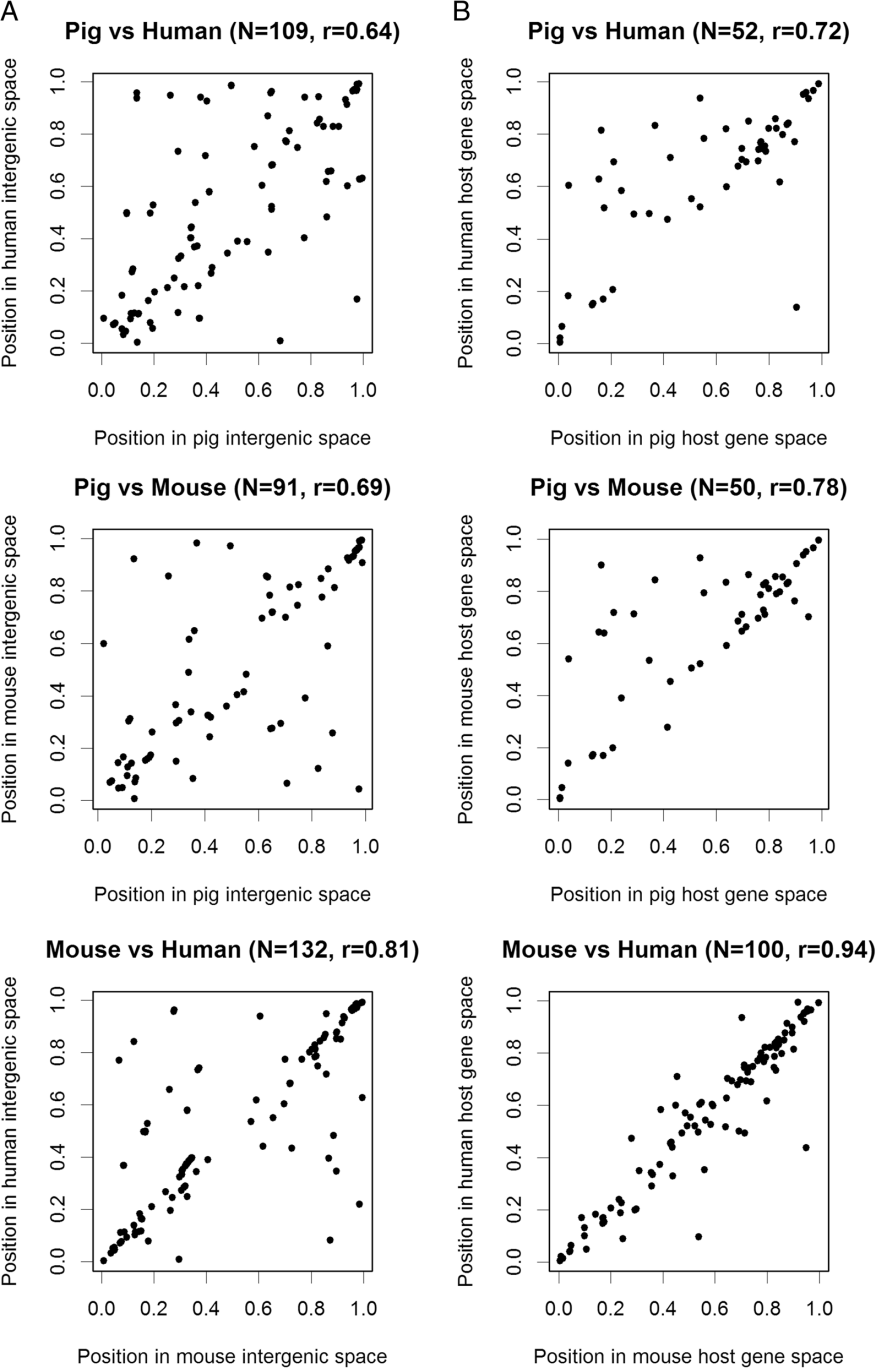
**Comparison of the position of miRNA genes in the intergenic regions and host genes (number of gene pairs and linear correlations are given in parantheses). A)** Positions of miRNA genes in intergenic space. The space between flanking protein-coding genes was standardized to 1 and the position of each intergenic miRNA gene was mapped on the standardized space. **B)** Positions of intragenic miRNA genes in gene space. The space between start and end of a host gene was standardized to 1 and the position of each intragenic miRNA gene was mapped on the standardized space.

We also examined precise location of intergenic miRNAs in intergenic space and of intragenic miRNA genes within host genes (Figure 3). In this analysis we required that only miRNA genes are one-to-one orthologues whereas flanking genes and host genes were not checked. The comparison revealed a considerable variation in miRNA position between species. The dissimilarities were present in all the three between-species comparisons and were slightly greater for intergenic miRNAs than for intragenic sequences. Some of the differences may result from existence of sequence repeats. We further analysed in greater detail the situations where orthologues miRNA genes have different location in two species (more than 0.25 in standardized space). In the pig-to-human comparison we observed 31 swine intergenic and 10 intragenic miRNA genes showing different position than their human orthologs.

With respect to the 31 intergenic miRNA genes, 11 of them also showed dissimilar location in pig-to-mouse comparison. Whereas average intergenic space is ~1 Mb, the 11 miRNA genes are located in much longer intergenic regions (average size ~4 Mb) and six of them are clustered witin a single region of ~6 Mb. Interestingly, in mouse-to-human comparison dissimilar locations (difference = 0.1, 28 mouse miRNA genes) were linked to shorter intergenic space (avg. ~ 0.6 Mb for 28 mice miRNA genes vs. ~1 Mb for random intergenic miRNA genes).

Concerning the 10 intragenic miRNA genes with dissimilar locations in pig-to-human comparison we also observed that 7 of them show dissimilar locations in pig-to-mouse comparison as well. The 7 miRNA genes are hosted in protein-coding genes that are not considerable longer than random pig host gene. However, we noticed that these dissimilar locations of intragenic miRNA genes can be explained by variation in number and size of introns in the two orthologues host genes, multiple transcription variants of protein coding gene, or by the fact that some UTR sequences are identified in human genome but not in the pig. Again, in mouse-to-human comparison dissimilar locations (difference = 0.1, 14 mouse miRNA genes) were linked to shorter host genes (avg. ~ 64 kb for 14 hosts vs. ~ 0.61 Mb for random hosts).

In conclusion, our comparisons suggest that positions of miRNA genes, relative to protein-coding genes, are conservative among studied mammal species. The orthologues intergenic miRNA genes are usually located within corresponding intergenic fragments being flanked by orthologues protein-coding genes. Similarly, the orthologues intragenic miRNA genes are hosted by protein-coding genes being orthologues as well. However, some number of dissimilar locations of orthologues miRNA genes cannot be excluded. Despite this conservatism, there are however differences in precise location of miRNAs in particular intergenic regions and within particular hosts.

### Pig-specific miRNA genes

Next we examined 463 porcine miRNA genes that have no orthologues in any of the genomes included in the Compara database. Such miRNAs are probably pig specific, however, it is also possible that for some miRNAs orthologues sequences exist but have not been discovered yet. Nevertheless this group of miRNA genes is probably phylogenetically young and we were interested in which part of the pig genome the new sequences evolved. Within the pig-specific miRNA genes, 67% was intergenic, 25% was intronic and 8% was exonic. These proportions are very similar to those calculated for all miRNA genes. This suggests that phylogenetically new intragenic miRNA genes evolve with the same frequency like phylogenetically old genes and that this proportion does not change in evolutionary time.

**Figure 4:**
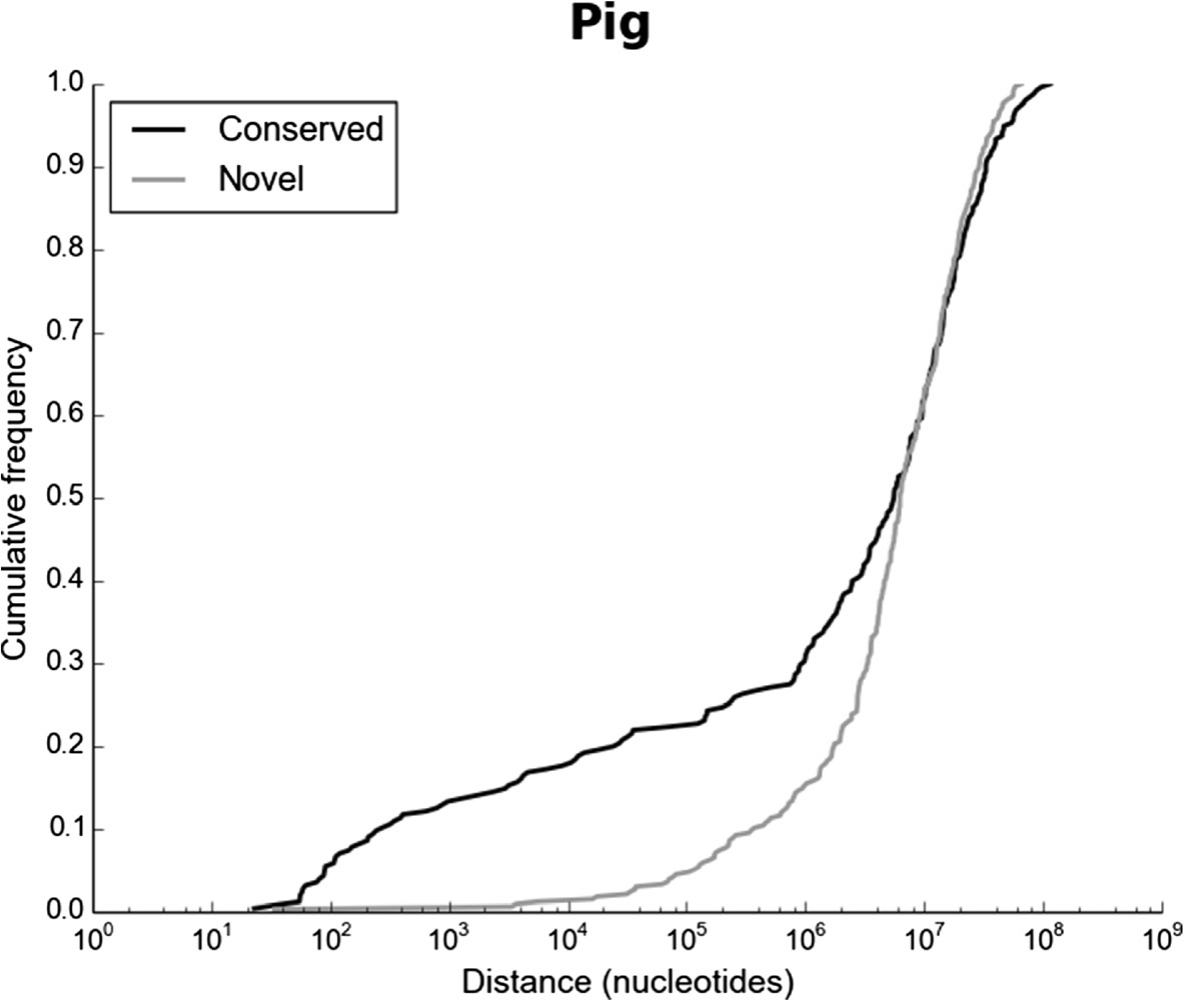
**Cumulative distance distribution of miRNA genes in pig.** Novel genes are pig-specyfic (based on Compara database). The distances (in nucleotides) between every two same-chromosome same-strand successive miRNA genes were obtained from Ensembl (ver. 77). Distance is drawn on a logarithmic scale.

We observed, however, that pig-specific miRNA genes (novel genes) are less likely to be clustered than conserved ones (Figure 4). We defined 3000 nt as the maximal distance for two same-chromosome same-strand miRNA genes to be considered as clustered [13]. The same definition was applied for inter- and intragenic miRNA genes. By this definition, porcine miRNAs genes are organized in 50 clusters, mostly pairs (34) and triplets (7). Within the pig-specific miRNA genes only 4% were found in clusters, whereas within the conserved genes up to 37% are organized in clusters. Similar tendency was observed for human-specific (9% and 37%, respectively) and mouse-specific miRNA genes (14% and 43%). This implies that phylogenetically new miRNA genes more like evolve in new chromosomal locations.

We considered all 95 pig-specific miRNA genes located within protein-coding genes on host strand. These 95 miRNAs genes were located in 94 protein-coding hosts. We identified all 67 human ortologs of these hosts (only one-to-one type was considered) and performed gene enrichment analyses. For this set of 67 genes the DAVID algorithm showed similar enrichment in gene-terms as for all previously considered hosts in the human genome. We conclude that phylogenetically young intragenic swine miRNA genes are not linked with any particular biological process via their host genes.

In order to search for pig-specific miRNA genes that are differentially expressed from other porcine miRNA genes we utilized publically available GEO datasets GSE28140. The data include miRNA expression evaluated using RAKE coupled with a spike-in based quantization method in 14 different swine tissues [20]. Within the group of the 463 pig-specific miRNA genes we identified 16 genes represented in the expression study, whereas the remaining group was represented by 96 miRNA. The expression data and the Mann–Whitney *U* test provided no evidence that pig-specific miRNA genes are differentially expressed in any of the tested porcine tissues. Across-tissue analysis revealed that transcript level of the pig-specific miRNA genes is slightly lower (p-value = 0.035), the difference, however, was very small and due to a few extreme values.

In previous studies striking positive correlation was found between expression levels of miRNA families and their age [12,21]. This observation concerns, however, longer evolutionary distances, for example, when primate-specific miRNA gene families are contrasted with ancient families. We found no evidence of differentially expressed miRNAs when pig-specific miRNAs are contrasted with the remaining genes. The comparison, however, included small number of pig-specific miRNA genes (n = 16), therefore is not definitive.

## Conclusions

Recent selective sweep analysis indicates that genes involved in RNA splicing and RNA processing may be under positive selection in pig lineage [6]. Here we studied pig miRNA - other key regulators of genes at the level of RNA. The distribution of miRNA genes in pig genome shows no particular relation to protein coding genes. Number of miRNA genes localized in intergenic regions, introns and exons roughly agrees with the size of these regions in pig genome. Similarly, a random distribution of miRNAs genes with no connection to the localization of protein-coding genes was observed here in human and mouse. The finding by other authors that miRNAs are more often localized in intragenic regions is true when both DNA strands are analysed together, but not separately.

We showed in human data that host genes harbouring intragenic miRNAs are not different from other protein-coding genes with respect to GO annotation. Similar result can be expected for the pig. Therefore we speculate that mechanism of posttranscriptional regulation of genes by their intragenic miRNAs is not limited to particular metabolic pathways and intragenic miRNAs are players in regulatory mechanisms for genes showing different pattern of expression. We observed that regions coding for pig pre-miRNAs have similar sequence to their human and mouse orthologues. Our further analysis showed that localization of pig miRNA genes and their orthologues in human and mouse is also similar. There are, however, differences in precise location of miRNAs in particular intergenic regions and within particular hosts. Whether these dissimilarities translates to dissimilar function or alters coexpression with protein-coding genes remains unknown. The most prominent difference between swine and human miRNAs is a large group of pig-specific sequences (53% of swine miRNAs). We found no evidence that this group of evolutionary new swine miRNAs is different from old miRNAs genes with respect to localisation in the pig genome except that they are less likely to be clustered. Functional studies of these miRNAs in future may reveal limits of the pig as a model organism to study human gene expression.

## Methods

The following species were included: pig (Sus scrofa), mouse (Mus musculus) and human (Homo sapiens). Annotations of miRNA sequences and protein coding genes were obtained from Ensembl genome databases, release 77 (October 2014) [22]. Ensembl release 77 included the high-coverage Sscrofa10.2 assembly of the pig genome (August 2011) produced by the Swine Genome Sequencing Consortium (SGSC). It consists of 20 chromosomes (1–18, X and Y) and 4562 unplaced scaffolds (GenBank assembly accession GCA_000003025.4). Data were retrieved and processed by the use of Ensembl Perl API (Ensembl Core Perl API for release 77). Protein coding genes were retrieved by the annotation keyword “protein_coding”, whereas miRNA sequences were retrieved by “miRNA” keyword.

To define inter- or intragenic miRNA location the variables *strand* (+/−), *chromosome number*, *start* and *end* positions for protein coding genes and miRNA sequencing were retrieved from Ensembl Database. A miRNA sequence was considered as intragenic when its entire length was included between *start* and *end* position of a protein coding gene, on the same *chromosome.* We observed two types of intragenic miRNA sequences: (a) located on same strand as host gene and (b) on opposite strand. Non-intragenic miRNAs were treated as intergenic sequences. Total genome length was taken directly from Ensembl summary of species genome. Proportion of gene sequences was calculated as the total length of all exons and introns (both strands) divided by total genome length in base pairs. The number of exons within a gene was obtained by a use of Ensembl Perl API function *get_all_exons,* which returns the number of all variants of each known exon. Therefore the total number of exons can be higher than transcribed.

CpG islands were predicted within a 5′ flanking region of 2 kbp. We use *newcpgreport* from Emboss package [23]. By default, this program defines a CpG island as a region where, over an average of 10 windows, the calculated % composition is over 50% and the calculated Obs/Exp ratio is over 0.6 and the conditions hold for a minimum of 200 bases. Codon usage statistic was calculated by programme *chips* from Emboss package (Nc statistics). For sequence comparison orthologues pairs of miRNAs were obtained from Ensembl database (by using Perl API Compara). After defining orthology the mature miRNA sequences were retrieved from miRBase. Sequences were aligned with the *water* (immature sequences) and *needle* (mature sequences) programmes from *Emboss* package. We applied the Database for Annotation, Visualization and Integrated Discovery (DAVID) ver. 6.7 and to identify enriched biological terms, including GO terms [24]. A term was considered important when 3 criteria were met: p-value (EASE) was below 0.01, percentage of user’s input gene hitting a given term was above 15% (but no less than 10 genes), and fold enrichment was ≥1.5.

## Additional files

Additional file 1: Figure S1. Distribution of location of intragenic miRNA genes in the genome of rat. The space between start and end of a host gene was standardized to 1 and the position of each intragenic miRNA gene was mapped on the standardized space. The number of miRNA genes analyzed are given in parentheses. Additional file 2: Data S1. Pig miRNA genes showing dissimilar locations in pig-to-human comparison.
