# Supplementary figures and images for: Distribution of miRNA genes in the pig genome
Source: BMC Genet. 2015 Jan 30;16(1):6. doi: 10.1186/s12863-015-0166-3 (PMC4318388; doi:10.1186/s12863-015-0166-3)

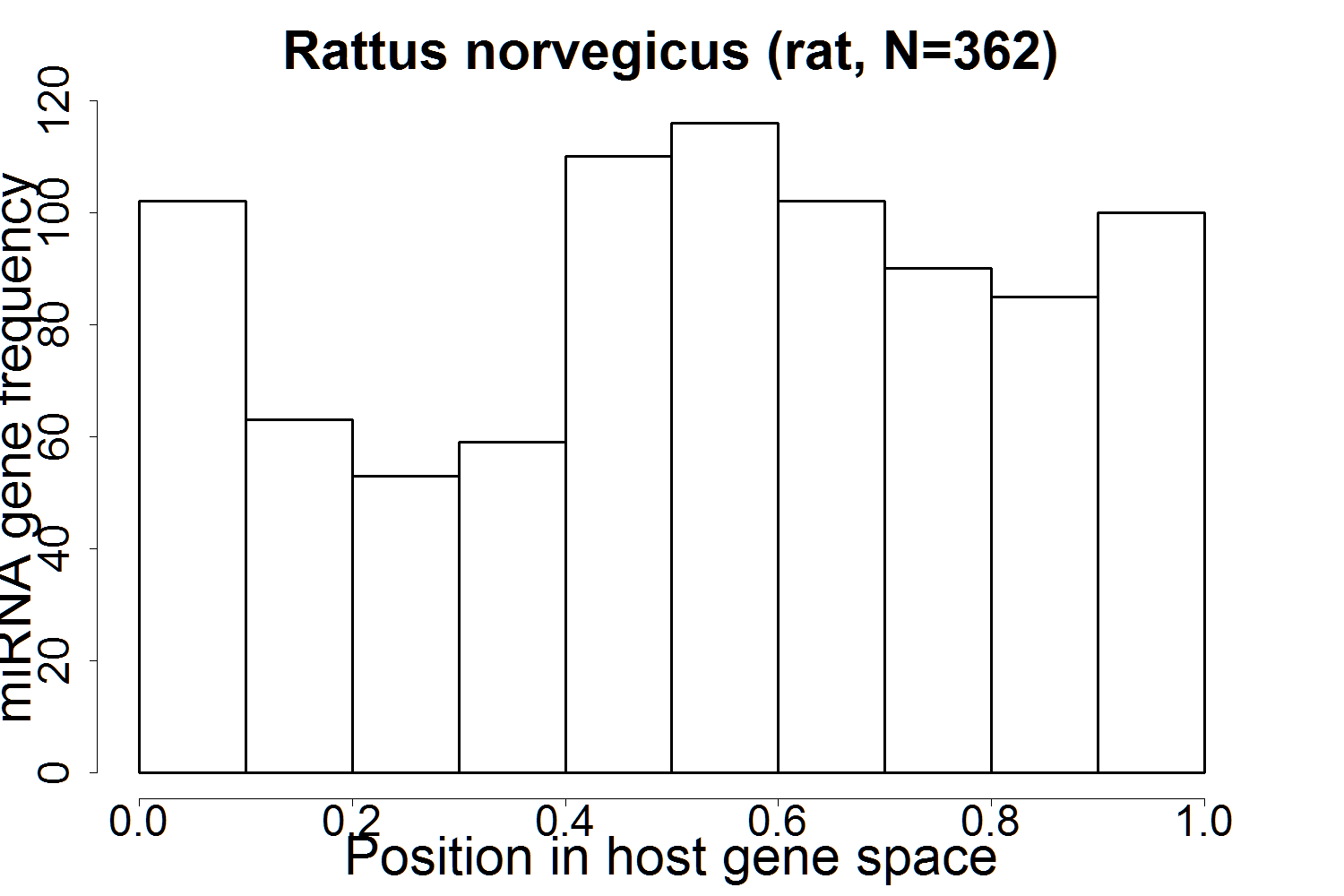

Supplement: Additional file 1: Figure S1. — Distribution of location of intragenic miRNA genes in the genome of rat. The space between start and end of a host gene was standardized to 1 and the position of each intragenic miRNA gene was mapped on the standardized space. The number of miRNA genes analyzed are given in parentheses. [file 12863_2015_166_MOESM1_ESM.tiff]
