# Supplementary material for: Distribution of miRNA genes in the pig genome
Source: BMC Genet. 2015 Jan 30;16(1):6. doi: 10.1186/s12863-015-0166-3 (PMC4318388; doi:10.1186/s12863-015-0166-3)
Supplement: Additional file 2: Data S1. — Pig miRNA genes showing dissimilar locations in pig-to-human comparison. [file 12863_2015_166_MOESM2_ESM.docx]

**Pig miRNA genes showing dissimilar location in pig-to-human comparison.**Annotations of miRNA sequences and protein coding genes were obtained from Ensembl genome databases, release 77 (October 2014)

**Six pig intragenic miRNA genes having one-to-one intergenic orthogues in human genome.**

ENSSSCG00000018838

ENSSSCG00000018414

ENSSSCG00000021735

ENSSSCG00000019354

ENSSSCG00000018754

ENSSSCG00000030659

**Ten pig intragenic miRNA genes having one-to-one orthologues in human genome being hosted by non-orthologues protein-coding genes.**

ENSSSCG00000019110

ENSSSCG00000019907

ENSSSCG00000019596

ENSSSCG00000018264

ENSSSCG00000019773

ENSSSCG00000019641

ENSSSCG00000030341

ENSSSCG00000019090

ENSSSCG00000019430

ENSSSCG00000029767

**Nine pig intergenic miRNA genes having human orthologues located in different intergenic regions**

ENSSSCG00000019393

ENSSSCG00000019658

ENSSSCG00000023071

ENSSSCG00000018271

ENSSSCG00000027451

ENSSSCG00000024128

ENSSSCG00000026678

ENSSSCG00000018206

ENSSSCG00000018867

**Three pig intergenic miRNA genes having human orthologues being intragenic**

ENSSSCG00000019246

ENSSSCG00000030509

ENSSSCG00000019942
